# *Mir142* loss unlocks IDH2^R140^-dependent leukemogenesis through antagonistic regulation of *HOX* genes

**DOI:** 10.1038/s41598-020-76218-8

**Published:** 2020-11-10

**Authors:** A. Marshall, J. Kasturiarachchi, P. Datta, Y. Guo, E. Deltcheva, C. James, J. Brown, G. May, N. Anandagoda, I. Jackson, J. K. Howard, E. Ghazaly, S. Brooks, A. Khwaja, M. Araki, K. Araki, D. Linch, G. M. Lord, T. Enver, R. Nimmo

**Affiliations:** 1 UCL Cancer Institute, University College London, London, UK; 2 School of Immunology and Microbial Sciences, King’s College London, London, UK; 3 School of Life Course Sciences, King’s College London, London, UK; 4 Centre for Haemato-Oncology, Barts Cancer Institute, Queen Mary University of London, London, UK; 5 Institute of Resource Development and Analysis, Kumamoto University, Kumamoto, Japan; 6 Faculty of Biology, Medicine and Health, University of Manchester, Manchester, UK; 7 Present Address: Medicines and Healthcare Products Regulatory Agency (MHRA), London, UK; 8 Present Address: Oxford Biomedica (UK) Ltd, Windrush Court, Transport Way, Oxford, OX4 6LT UK; 9 Present Address: The Institute of Cancer Research, London, UK

**Keywords:** Cancer models, Leukaemia, Experimental models of disease, Cancer models

## Abstract

AML is a genetically heterogeneous disease and understanding how different co-occurring mutations cooperate to drive leukemogenesis will be crucial for improving diagnostic and therapeutic options for patients. *MIR142* mutations have been recurrently detected in IDH-mutated AML samples. Here, we have used a mouse model to investigate the interaction between these two mutations and demonstrate a striking synergy between *Mir142* loss-of-function and IDH2^R140Q^, with only recipients of double mutant cells succumbing to leukemia. Transcriptomic analysis of the non-leukemic single and leukemic double mutant progenitors, isolated from these mice, suggested a novel mechanism of cooperation whereby *Mir142* loss-of-function counteracts aberrant silencing of *Hoxa* cluster genes by IDH2^R140Q^. Our analysis suggests that IDH2^R140Q^ is an incoherent oncogene, with both positive and negative impacts on leukemogenesis, which requires the action of cooperating mutations to alleviate repression of *Hoxa* genes in order to advance to leukemia. This model, therefore, provides a compelling rationale for understanding how different mutations cooperate to drive leukemogenesis and the context-dependent effects of oncogenic mutations.

## Introduction

Acute myeloid leukemia (AML) arises from the accumulation of mutations in hematopoietic stem or progenitor cells resulting in the formation of an aberrant leukemic clone with defective differentiation. The expansion of this clone leads to impaired hematopoiesis and, subsequently, bone marrow failure. AML is a heterogeneous disease and a large number of recurrently mutated genes have been identified, with most leukemia cells carrying at least two driver mutations, but it is not known how these mutations collaborate to drive leukemogenesis^1^.

One set of frequently mutated genes are the isocitrate dehydrogenase (IDH) genes. Mutations in either IDH1 or IDH2 are common in AML, found in about 20% of cytogenetically normal patients, and occur at three particular residues—R132 in IDH1, and R140 or R172 in IDH2. The mutations at these residues disrupt the ability of the IDH enzymes to convert isocitrate to α-ketoglutarate^2^, and, importantly, display neomorphic activity, resulting in the aberrant production of high levels of the oncometabolite R-2-hydroxyglutarate (2-HG), which inhibits the function of dioxygenases. α-ketoglutarate-dependent dioxygenases are a broad group which includes epigenetic modifiers such as the TET family of enzymes, involved in DNA demethylation, and Jumonji-domain-containing histone demethylases^3–6^.

In the Cancer Genome Atlas (TGCA) AML cohort, four patients out of 200 were identified with mutations in the miRNA *MIR142*, all of which were exclusively identified in IDH-mutated AML samples, suggesting a strong genetic cooperation between these two types of mutation^1^. Three patients had an IDH2^R140^ mutation while one had an IDH1^R132^ mutation. In addition, *MIR142* was found to be recurrently mutated in another cohort of AML and MDS patients, as well as in some lymphomas^7,8^.

**Figure 1:**
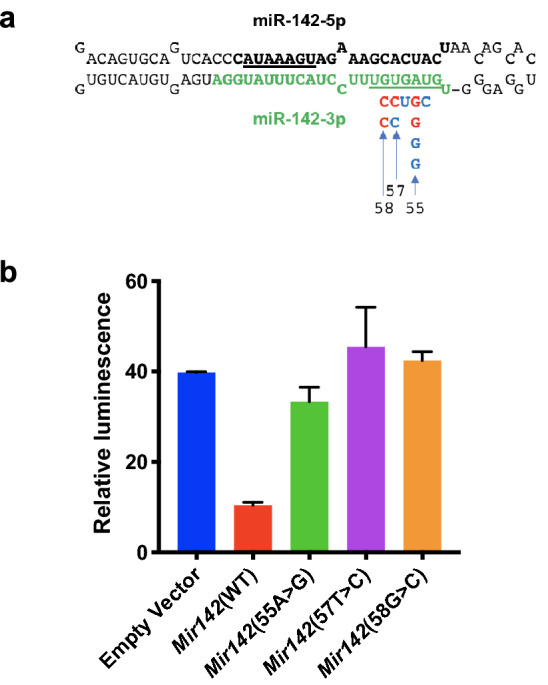
Mutations affecting the seed region of miR-142-3p identified in AML patients result in loss of function. (**a**) *Mir142* pre-miR hairpin showing position and nature of individual single nucleotide variants identified in AML and MDS patients. Numbering based on position in pre-miR sequence. Mature miRNA sequences shown in bold with seeds underlined. Wild type miR-142-3p is in green with mutations identified by TCGA in red and those identified by Thol et al. in blue. (**b**) Luciferase assay showing relative expression of a miR-142-3p reporter construct in cells transduced with empty vector (negative control), WT *MIR142* vector (positive control), or vectors expressing *MIR142* variants corresponding to each of the three TCGA point mutations—mut55 A > G, mut57 U > C and mut58 G > C. Numbering based on position in pre–miR sequence. Data shown as mean ± standard deviation of relative luminescence activity of firefly luciferase normalized to *Renilla* luciferase (*n* = 3).

*MIR142* is a highly conserved miRNA which is abundantly expressed, predominantly in the hematopoietic system, and which regulates the differentiation and function of multiple hematopoietic cell types including megakaryocytes^9^, CD4+ dendritic cells^10^, T cells^11–14^, and erythrocytes^15^. The mutations in *MIR142* found in AML and MDS were exclusively located in the miR-142-3p region (Fig. 1a), consistent with the observation that this is the predominant mature form of *MIR142* in hematopoietic cells^10,16^. Furthermore, all variants affected the seed sequence of miR-142-3p and are thus likely to disrupt the interaction between miR-142-3p and its cognate targets.

The striking co-incidence of mutant miR-142-3p with neomorphic IDH1/2 mutations strongly suggested that *MIR142* and IDH mutations are likely to exhibit a synergistic leukemogenic effect. A recent study investigated the potential interaction between these mutations, but synergy between IDH2^R172^ and loss of *Mir142* was obscured by the ability of IDH2^R172^ alone to drive the development of a fatal myeloid neoplasia^17^. However, the *MIR142* mutations identified in the TCGA cohort co-occurred with either IDH2^R140^ or IDH1^R132^, but not IDH2^R172^ (ref.^1^). IDH2^R172^ alleles have been suggested to be stronger than IDH2^R140^, with increased production of 2-HG^18,19^, which is consistent with the observation that mutations at this site do not significantly co-occur with other recurrent mutations, and IDH2^R172^ mutant-leukemias have been classified into a distinct genetic sub-group^20–23^. In contrast, IDH2^R140^ and IDH1^R132^ mutants often co-occur with other recurrent mutations, particularly NPM1^21,22^. Mouse models have revealed that IDH2^R140^ and IDH1^R132^ variants are insufficient to drive leukemogenesis on their own but are able to do so when introduced in combination with other oncogenes commonly associated with AML^24–26^. However, many of the mutations investigated in these studies are not reflective of those that naturally co-occur with mutant IDH in human leukemias and the mechanism underlying the requirement for cooperating mutations in these IDH-dependent leukemias has yet to be elucidated.

Here, we have investigated the mechanism of cooperation between mutations in two genes that are co-mutated in AML. We combined *Mir142* loss-of-function with a disease-relevant allele of *IDH2*, *IDH2*^*R140*^^*Q*^. This analysis revealed that *Mir142* loss-of-function unlocked the leukemogenic potential of IDH2^R140Q^, and uncovered an unanticipated negative impact of IDH2^R140Q^ upon expression of pro-leukemic *Hoxa* cluster genes in myeloid progenitors which was alleviated by *Mir142* loss-of-function, thus releasing their combined leukemogenic potential. This study highlights the possibility that oncogenic driver mutations such as IDH2^R140^, can act incoherently in leukemogeneis, with progression to leukemia being dependent upon the compensatory activity of a cooperating mutation to counteract their anti-leukemic effects.

## Results

### Mutations in miR-142-3p identified in AML cause loss of function

*MIR142* is the only miRNA found to be recurrently mutated in AML and, significantly, all the mutations so far identified were point mutations localized at multiple different positions within the seed region of miR-142-3p (Fig. 1a). Since the seed region of miRNAs is the major determinant for binding to cognate targets, it is likely that these mutations cause loss of function. To investigate this, we examined the effect of the variant forms of *MIR142* on the expression of a luciferase reporter containing three copies of a bulged miR-142-3p canonical binding site. HEK293T cells were transduced with lentiviral vectors expressing either WT *MIR142* or *MIR142* variant hairpins corresponding to each of the three TCGA point mutations—mut55 A > G, mut57 U > C and mut58 G > C (numbering based on position in the pre-miR hairpin as shown in Fig. 1a), or empty vector control. The activity of the luciferase reporter was reduced by WT *MIR142* expression, but the variant forms all failed to cause downregulation (Fig. 1b). These findings were consistent with those reported by others^8,17^ and demonstrated that the *MIR142* mutations identified in leukemia patients result in loss of function, providing a strong rationale for using *Mir142* knockout (KO) mice to investigate the role of *MIR142* mutations in AML etiology.

### *Mir142* loss-of-function synergises with IDH2^R140Q^ to promote leukemogenesis in a mouse model

To investigate whether *Mir142* loss-of-function and IDH2^R140^^Q^ have a synergistic leukemogenic effect, we used two strains of *Mir142* KO mice (Supplementary Fig. 1a,b). CRISPR-Cas9 genome editing was used to generate a specific 26 bp deletion of the region encoding miR-142-3p (*Mir142*^*em2Card*^*,* provided by Masatake and Kimi Araki, Centre for Animal Resources and Development (CARD), Kumamoto University, Japan), and we confirmed our findings using the *Mir142*^*HOJ2*^ strain which harbors a 276 bp deletion of the *Mir142* locus generated through germline Cre-mediated recombination of a *LoxP* targeted allele (provided by Graham Lord, King’s College London, UK, Supplemental Fig. 1a)^14^. Loss of *Mir142* expression was confirmed by qRT-PCR (Supplementary Fig. 1b). Both the *Mir142*^*HOJ2*^ and *Mir142*^*em2Card*^ strains exhibited the same phenotypes, and we refer to them collectively as *Mir142*^−/−^ mice. Consistent with the findings of Trissal et al., *Mir142*^−/−^ mice did not develop leukemia, demonstrating that *Mir142* loss-of-function alone is not sufficient for leukemogenesis^17^.

**Figure 2:**
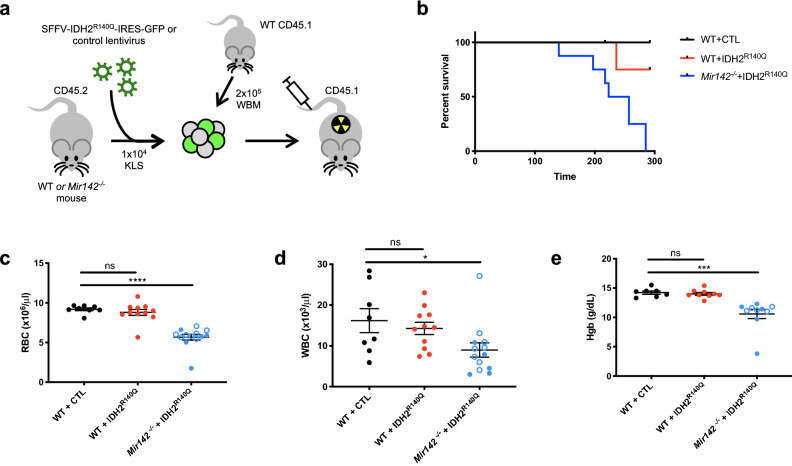
*Mir142* loss of function synergizes with IDH2^R140^^Q^ to promote myeloid leukemogenesis in mice. (**a**) Schematic diagram summarizing the model. KLS: c-Kit^+^ Lin^-^ Sca1^+^ cells. (**b**) Kaplan–Meier analysis of survival of mice transplanted with WT + CTL (empty vector control, black, *n* = *5*), WT + IDH2^R140Q^ (red, *n* = *7*) or *Mir142 *^–/–^ + IDH2^R140Q^ (blue, *n* = *8*) HSPCs. Mantel-Cox log–rank test: *Mir142 *^–/–^ + IDH2^R140Q^ versus WT CTL *P* = *0.0308*, *Mir142 *^–/–^ + IDH2^R140Q^ versus WT + IDH2^R140Q^ *P* = *0.0134*, and WT + IDH2^R140Q^ versus WT CTL *P* = *0.617* (not significant). Survival data from 2 independent experiments using the two different *Mir142*^–/–^ strains. (**c**,**d**) Red blood cell (RBC), (**c**) and white blood cell counts (WBC), (**d**) from recipients of transduced HSPCs, analyzed 4–6 months after transplantation (WT + CTL *n* = *8*, WT + IDH2^R140Q^ *n* = *11*, *Mir142 *^–/–^ + IDH2^R140Q^ *n* = *13*). (**e**) Hemoglobin levels in peripheral blood of transplant recipients 4–6 months after transplantation (WT + CTL *n* = *7* , WT + IDH2^R140Q^ *n* = *9*, *Mir142 *^–/–^ + IDH2^R140Q^ *n* = *10*). Data shown in c-e pooled from 3–4 independent experiments, using both *Mir142*^–/–^ strains (*Mir142*^*em2Card*^: open blue circles. *Mir142*^*HOJ2*^*:* filled blue circles).

To investigate whether IDH2^R140^ mutants cooperate with *Mir142* loss-of-function to promote leukemic transformation, we introduced the IDH2^R140Q^ gene into *Mir142*^−/−^ and wild-type hematopoietic stem/progenitor cells (HSPCs) using a lentiviral construct co-expressing the oncogene together with a GFP reporter (Fig. 2a, Supplementary Fig. 1c,d). FACS sorted CD45.2+ KLS HSPCs were transduced with SFFV-IDH2^R140Q^-IRES-GFP (IDH2^R140Q^) or SFFV-IRES-GFP control (CTL) vector and transplanted into irradiated CD45.1+ recipients with WT CD45.1 + whole bone marrow (BM) competitor cells (Fig. 2a). Consistent with the reported neomorphic function of IDH mutants, we observed high levels of 2-HG in IDH2^R140Q^-expressing BM cells (Supplementary Fig. 1e). Strikingly, all the recipients of *Mir142*^−/−^ cells transduced with IDH2^R140Q^ vector, (*Mir142*^−/−^ + IDH2^R140Q^ double mutants) became ill, suffering from severe cytopenia and anemia, and had to be culled (median survival = 8 months; Fig. 2b–e). In contrast, all control animals transplanted with WT cells transduced with CTL vector (WT + CTL), and the majority of recipients transplanted with WT cells transduced with IDH2^R140Q^ (WT + IDH2^R140Q^), remained healthy throughout the experiment. There was, therefore, a clear synergistic impact on survival resulting from the combination of *Mir142* loss-of-function with IDH2^R140Q^ compared to IDH2^R140Q^ alone.

### Impaired hematopoiesis and expansion of myeloid progenitors in *Mir142* KO mice

**Figure 3:**
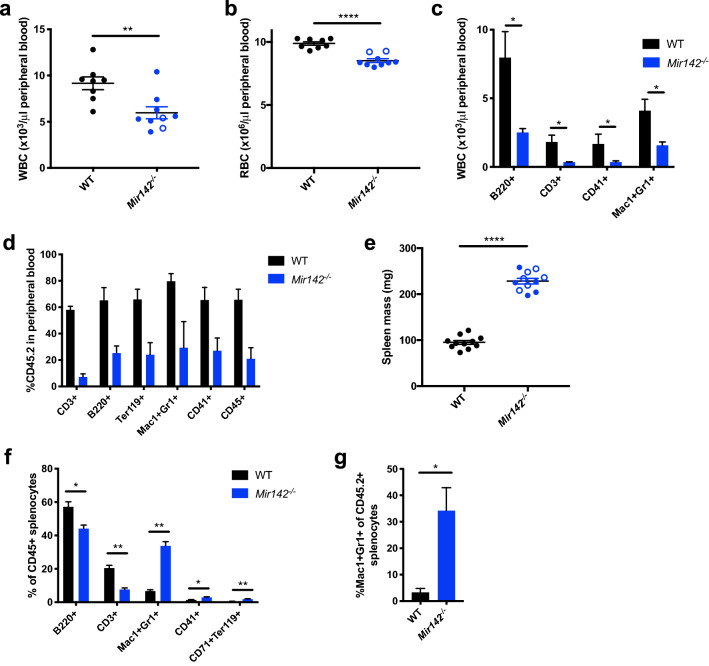
*Mir142* loss of function leads to impaired hematopoiesis and reduced hematopoietic reconstitution capacity. (**a**,**b**) WBC (**a**) and RBC (**b**) counts from WT and *Mir142*^–/–^ mice (WT *n* = *8*, *Mir142*^–/–^* n* = *9*). (**c**) Lineage distribution within peripheral blood WBCs (WT *n* = *4*, *Mir142*^–/–^* n* = *5*). (**d**) Proportion of CD45.2^+^ donor cells within total CD45 (CD45.1 + CD45.2) in peripheral blood at 3 months after transplantation of WT or *Mir142*^–/–^ HSPCs (WT *n* = *4*, *Mir142*^–/–^* n* = *3*). (**e**) Spleen size in WT and *Mir142*^–/–^ mice (*n* = *11* each). (**f**) Lineage distribution within CD45^+^ splenocytes from WT and *Mir142*^–/–^ mice: B cells (B220^+^), T cells (CD3^+^), granulocytes (Mac1^+^Gr1^+^), megakaryocytes (CD41^+^) and erythroid progenitors (CD71^+^Ter119^+^) (*n* = *5* each for WT and *Mir142*^–/–^). (**g**) Frequency of Mac1^+^Gr1^+^ myeloid cells within CD45.2^+^ donor cells in the spleens of recipients transplanted with WT or *Mir142*^–/–^ bone marrow cells (WT *n* = *5*, *Mir142*^–/–^*n* = *4*). Pooled data is shown in (**a**,**b**), and (**e)**, from analysis of both strains of *Mir142*^–/–^ mice (*Mir142*^*HOJ2*^* :* open blue circles. *Mir142*^*em2Card*^: filled blue circles). Data in (**c**,**g**) generated with *Mir142*^*HOJ2*^*,* and data in (**d**,**f**) generated with *Mir142*^*em2Card*^*.*

To understand the relative contributions of *Mir142* and IDH2 mutations to leukemogenesis, we examined the hematopoietic defects associated with loss of *Mir142* alone using our KO mouse models. Analysis of peripheral blood (PB) in *Mir142* KO mice revealed pan-cytopenia (Fig. 3a,b) arising from a reduction in all lineages (Fig. 3c). Transplantation of wild type or *Mir142*^−/−^ BM or HSPCs (CD45.2+) into irradiated CD45.1+ recipients revealed that *Mir142*^−/−^ HSCs exhibit reduced reconstitution capacity (Fig. 3d) suggesting that the defect is intrinsic to the hematopoietic compartment and not due to impairment of the BM niche in KO mice. *Mir142*^−/−^ mice also exhibited significant splenomegaly, with a doubling in spleen mass (Fig. 3e), and there was a large increase in the *Mir142*^−/−^ Mac1^+^Gr1^+^ myeloid population in the spleens of both the constitutive *Mir142* KO and the transplant recipients (Fig. 3f,g). Splenomegaly and extramedullary hematopoiesis can be associated with inefficient myelopoiesis and so may reflect a compensatory response to the reduction in circulating myeloid cells observed in the *Mir142*^−/−^ mice.

**Figure 4:**
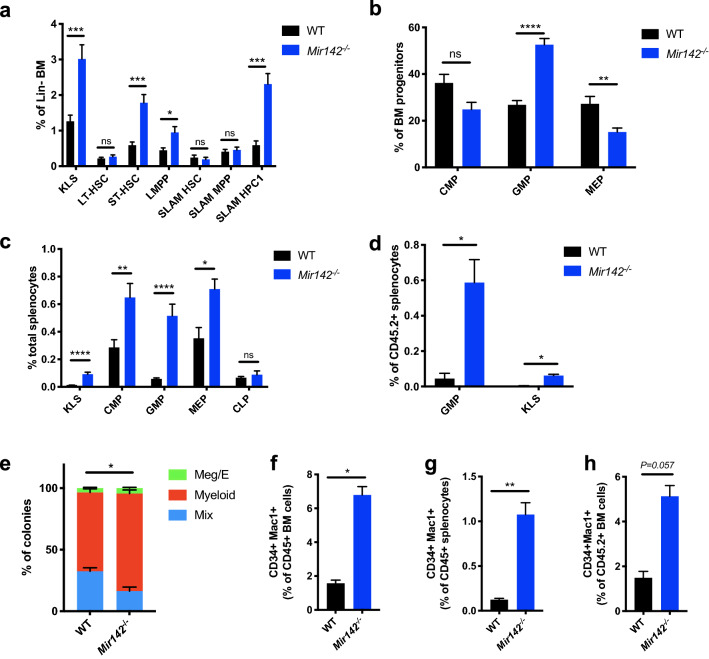
*Mir142* loss of function leads to expansion of myeloid progenitors and formation of an aberrantly expanded CD34^+^Mac1^+^ myeloblast population. (**a**) Proportion of HSC subsets within the bone marrow as assessed by CD34/Flt3 or SLAM markers (CD150/CD48) (WT *n* = *10*, *Mir142*^–/–^* n* = *9*). LT-HSC (CD34^–^Flt3^–^ KLS), ST-HSC (CD34^+^ Flt3^–^ KLS), LMPP (CD34^+^ Flt3^+^ KLS), SLAM HSC (CD150^+^ CD48^–^ KLS), SLAM MPP (CD150^–^ CD48^–^ KLS) and SLAM HPC1 (CD150^–^CD48^+^ KLS). (**b**) Proportion of myeloid progenitors within the bone marrow (WT *n* = *10*, *Mir142*^–/–^* n* = *9*). (**c**) Proportion of multipotent and committed progenitor populations in the spleen (WT *n* = *9*, *Mir142*^–/–^* n* = *8*). (**d**) Frequency of multipotent (KLS) and myeloid committed progenitors (GMPs) within CD45.2^+^ donor cells in the spleens of transplant recipients (WT *n* = *5*, *Mir142*^–/–^* n* = *4*). (**e**) Proportion of myeloid, megakaryocyte/erythroid (Meg/E) and mixed colonies in CFC assay from FACS sorted HSPCs (KLS). Pooled data shown from 4 independent experiments. Statistical significance shown for proportion of myeloid colonies. (**f**,**g**) Proportion of CD34^+^ Mac1^+^ cells within CD45^+^ bone marrow cells (**f**) and CD45^+^ splenocytes (**g**) (*n* = *5* each for WT and *Mir142*^–/–^). (**h**) Proportion of CD34^+^Mac1^+^ cells within CD45.2^+^ donor cells in the bone marrow of recipients transplanted with WT or *Mir142*^–/–^ HSPCs (WT *n* = *4*, *Mir142*^–/–^* n* = *3*). All data generated using *Mir142*^*em2Card*^ KO mice, except D, for which the *Mir142*^*HOJ2*^ strain was used.

The proportion of HSCs, defined as either CD34^−^Flt3^-^KLS (LT-HSC) or CD150^+^CD48^-^KLS (SLAM HSC), was not significantly impacted in the *Mir142*^−/−^ mice (Fig. 4a); however, there was a significant expansion of the KLS population, predominantly accounted for by increased numbers of multipotent progenitors defined as either CD34^+^Flt3^-^KLS (ST-HSC) or CD150^-^CD48^+^ (SLAM HPC1) progenitors (Fig. 4a). Analysis of the committed progenitor compartment revealed myeloid skewing in *Mir142*^−/−^ mice, with a significant increase in the proportion of GMPs, and a concomitant reduction of MEPs in the bone marrow of the KO mice (Fig. 4b). There was also a large increase in the number of GMPs in the spleens of the KO mice and within the donor-derived *Mir142*^−/−^ cells in the spleens of the transplant recipients (Fig. 4c,d). In addition, there was a significant increase in the proportion of myeloid colonies generated by *Mir142*^−/−^ HSPCs in CFC assays, consistent with the myeloid-bias observed in the progenitor populations in these animals (Fig. 4e).

However, the clearest abnormality was the presence of an aberrant myeloblast population in the bone marrow and spleens of the *Mir142*^−/−^ animals and in recipients transplanted with *Mir142*^−/−^ cells. This population co-expressed Mac1^+^, a marker of committed myeloid cells, and CD34, a protein normally confined to primitive hematopoietic stem and progenitor cells, suggesting a partial block in myeloid differentiation (Fig. 4f–h) consistent with the reduction in circulating Mac1^+^Gr1^+^ cells in the *Mir142*^−/−^ mice. Increased frequency of myeloblasts is a defining feature of AML, and the presence of this expanded CD34^+^ Mac1^+^ aberrant myeloid population in *Mir142*^−/−^ animals strongly supported a role for *Mir142* loss-of-function in promoting leukemogenesis; however, this population never rose above 10% of CD45^+^ cells in the bone marrow, consistent with the failure of *Mir142* loss-of-function to promote leukemia in the absence of IDH2^R140^^Q^.

### Cooperation between *Mir142* loss-of-function and IDH2^R140Q^ drives leukemic transformation of myeloid progenitors

**Figure 5:**
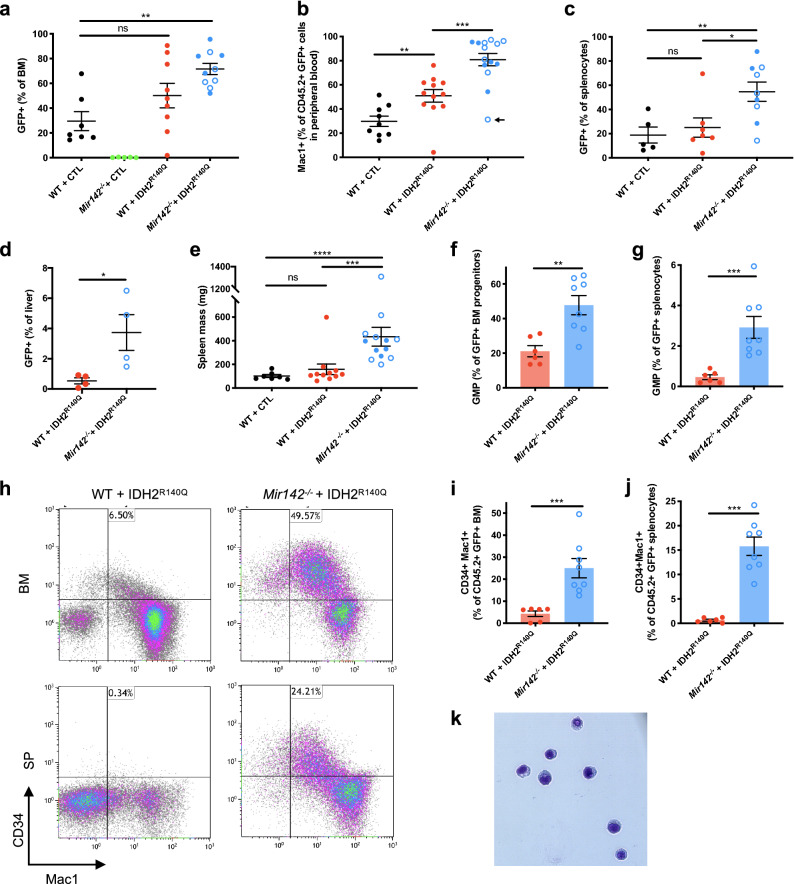
Expansion of myeloid progenitors and CD34^+^Mac1^+^ myeloblasts in *Mir142*^–/–^ + IDH2^R140^^Q^ leukemias. (**a**) Proportion of GFP^+^ cells in the bone marrow from recipients of transduced HSPCs (WT + CTL *n* = *7*, *Mir142*^–/–^ + CTL *n* = *5*, WT + IDH2^R140Q^ *n* = *9*, *Mir142*^–/–^ + IDH2^R140Q^ *n* = *10*). (**b**) Proportion of Mac1^+^ cells within CD45.2^+^GFP^+^ population in peripheral blood from recipients of transduced HSPCs (WT + CTL *n* = *9*, WT + IDH2^R140Q^ *n* = *12*, *Mir142 *^–/–^ + IDH2^R140Q^ *n* = *14*). Arrow indicates an animal which had a lower proportion of Mac1 + cells due to the majority (66%) of CD45.2^+^GFP^+^ peripheral blood cells having a more primitive CD34^+^Mac1^–^ phenotype. (**c**) Percentage GFP^+^ cells in spleens from recipients of transduced HSPCs (WT + CTL *n* = *5*, WT + IDH2^R140Q^ *n* = *7*, *Mir142 *^–/–^ + IDH2^R140Q^ *n* = *9*). (**d**) Proportion of GFP^+^ cells in the livers from recipients of transduced HSPCs (WT + IDH2^R140Q^ *n* = *4*, *Mir142 *^–/–^ + IDH2^R140Q^ *n* = *4*). (**e**) Spleen size in recipients of transduced HSPCs (WT + CTL *n* = *7,* WT + IDH2^R140Q^ *n* = *11*, *Mir142 *^–/–^ + IDH2^R140Q^ *n* = *13*). (**f**,**g**) Proportion of GMPs within GFP^+^ progenitors (c-Kit^+^ Lin^–^ Sca1^–^ compartment) in the bone marrow (**f**) or spleens (**g**) of recipients of transduced HSPCs (WT + IDH2^R140Q^ *n* = *6*, *Mir142*^–/–^ + IDH2^R140Q^ *n* = *8*). (**h**) Representative FACS plots showing proportion of CD34^+^ Mac1^+^ myeloblasts within the GFP^+^ population in the bone marrow (BM, top panels) or spleen (SP, lower panels) from recipients of transduced HSPCs. (**i**,**j**) Proportion of CD34^+^ Mac1^+^ myeloblasts within the GFP^+^ population in the bone marrow (**i**) or spleen (**j**) from recipients of transduced HSPCs (WT + IDH2^R140Q^ *n* = *6*, *Mir142*^–/–^ + IDH2^R140Q^ *n* = *8*). (**k**) Cytospin of *Mir142*^–/–^ + IDH2^R140Q^ CD34^+^Mac1^+^ cells showing myeloblastic morphology. Data in (**a**–**c**,**e**) is pooled from 3 to 4 independent experiments using both *Mir142*^*em2Card*^ (open circles) and *Mir142*^*HOJ2*^ (filled circles) KO mice. Data in (**f**–**j**) generated using only the *Mir142*^*em2Card*^ allele, pooled from 2 independent experiments.

To investigate how *Mir142* and IDH^R140^ mutations synergize to drive leukemogenesis, we further explored the effect of IDH2^R140Q^ in the presence or absence of *Mir142*. There was a significant increase in the proportion of GFP^+^ donor cells in the bone marrow of *Mir142*^−/−^ + IDH2^R140Q^ recipients compared to the recipients of wild type cells transduced with empty vector (WT + CTL), such that the majority of cells in the bone marrow of the double-mutant recipients were GFP^+^, demonstrating that the combination of *Mir142* loss-of-function and IDH2^R140Q^ provides a strong competitive advantage (Fig. 5a). In contrast, there was no significant increase in the frequency of GFP^+^ cells in the recipients of single mutant WT + IDH2^R140Q^ cells. Notably, *Mir142*^−/−^ + CTL HSPCs failed to engraft (< 1% donor chimerism) precluding further analysis, but transduction of *Mir142*^−/−^ cells with IDH2^R140Q^ rescued their defective engraftment and allowed their expansion in the recipients, resulting in the development of leukemia (Fig. 5a). Therefore, the IDH2^R140Q^ mutant compensated for a detrimental effect of *Mir142* loss-of-function in this setting.

In line with the synergistic impacts seen on survival and on inhibition of normal hematopoiesis (severe cytopenia and anemia) (Fig. 2b–e), the *Mir142*^−/−^ + IDH2^R140^^Q^ recipients displayed an increased proportion of Mac1^+^ myeloid cells in the peripheral blood, consistent with myeloid neoplasia (Fig. 5b). Furthermore, GFP^+^ leukemia cells infiltrated into the spleen and liver (Fig. 5c,d), and double mutant (*Mir142*^−/−^ + IDH2^R140Q^) recipients exhibited more severe splenomegaly compared to both the single mutant (WT + IDH2^R140Q^) recipients (Fig. 5e) and the *Mir142* knockout alone (Fig. 3e).

AML is a heterogeneous disease characterized by a block in myeloid differentiation and expansion of aberrant myeloid progenitors. Many studies have demonstrated that AML may arise from transformation of committed myeloid progenitors^27–30^. In the leukemias generated by *Mir142*^−/−^ + IDH2^R140^^Q^ cells, the majority of the lineage negative, c-Kit^+^ progenitor cells in the bone marrow had a GMP-like phenotype, and the proportion of GMPs was significantly increased in the *Mir142*^−/−^ + IDH2^R140Q^ double mutants compared to the single IDH2^R140Q^ mutants (48% compared to 21% of GFP^+^ BM progenitor cells) (Fig. 5f), with a particularly large increase in the frequency of GMPs within the spleen (Fig. 5g). In addition, there was a very large (~ sixfold) expansion in the number of CD34^+^Mac1^+^ myeloblasts in the leukemic *Mir142*^−/−^ + IDH2^R140Q^ recipients (Fig. 5h–j) compared to single mutant WT + IDH2^R140Q^ recipients (from 4% to 25% of GFP^+^CD45.2^+^ BM cells). This was also significantly higher than observed for *Mir142* loss-of-function alone, in either steady state or transplantation settings (Fig. 4f–h). Cytospins of FACS-purified CD34^+^Mac1^+^ cells confirmed that this population was composed of primitive myeloid cells with a myeloblastic morphology (Fig. 5k). This analysis therefore revealed a strong synergistic impact of *Mir142* loss-of-function and IDH2^R140Q^ in promoting expansion of immature myeloid progenitor populations, underlying their cooperative leukemogenic effect.

### *Mir142* loss-of-function antagonizes IDH2^R140Q^-dependent silencing of *Hoxa* cluster genes in myeloid progenitors

**Figure 6:**
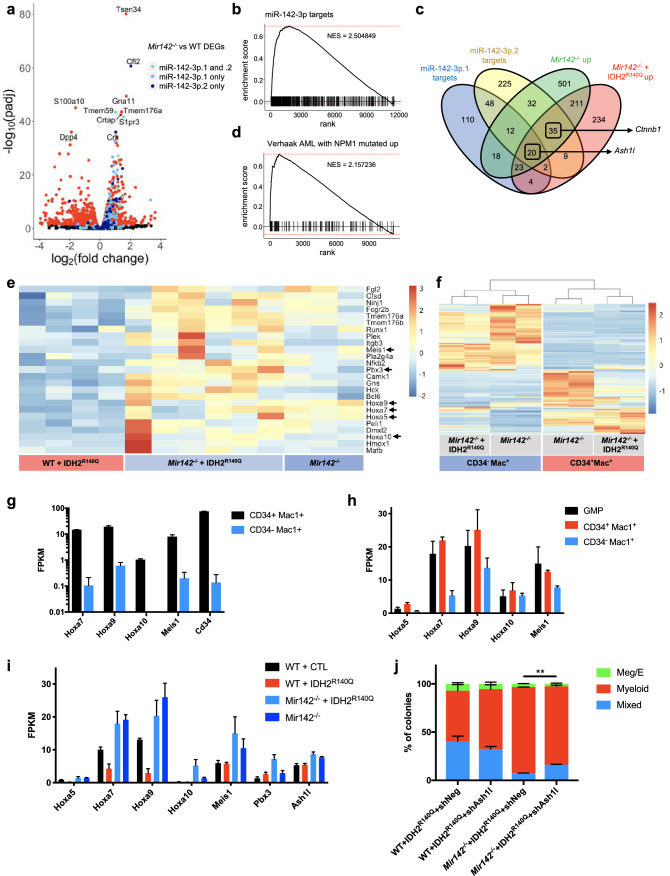
*Mir142* loss of function upregulates a leukemic *HOX/Meis1/Pbx3* signature and antagonizes IDH2^R140^^Q^-dependent silencing of *Hoxa* cluster genes. (**a**) Volcano plot showing differentially expressed genes (DEGs) in *Mir142*^–/–^ versus WT GMPs (*padj* < 0.05 colored dots, WT *n* = 4, *Mir142*^–/–^ *n* = 3). miR-142-3p targets predicted by Targetscan highlighted in blue. Light blue: miR–142–3p.1 specific targets. Dark blue: miR-142-3p.2 specific targets. Turquoise: targets of both miR-142-3p.1 and miR-142-3p.2 isomirs. Top ten most significantly differentially expressed genes are labelled. (**b**) Gene set enrichment analysis (GSEA) reveals strong enrichment of predicted miR-142-3p targets (union of miR-142-3p.1 and miR-142-3p.2 targets) in genes upregulated in *Mir142*^–/–^ GMPs compared to WT. (**c**) Venn diagram showing overlap of miR-142-3p.1 and miR-142-3p.2 targets with DEGs from *Mir142*^–/–^ versus WT GMPs and *Mir142*^–/–^ + IDH2^R140Q^ versus WT + IDH2^R140Q^ GMPs (all DEGs with *padj* < 0.05 were used for the analysis). Highlighted targets *Ash1l* and *Ctnnb1* are differentially expressed in both *Mir142*^–/–^ and *Mir142*^–/–^ + IDH2^R140Q^ GMPs. (**d**) GSEA reveals strong enrichment of a mutant NPM1–associated leukemic signature in genes upregulated in *Mir142*^–/–^ + IDH2^R140Q^ GMPs compared to WT + IDH2^R140Q^. (**e**) Heatmap showing expression of the significantly differential leading-edge genes contributing to enrichment of the mutant NPM1–associated leukemic signature in *Mir142*^–/–^ + IDH2^R140Q^ verus WT + IDH2^R140Q^ GMPs (*padj* < *0.05*). Signature includes multiple homeobox family genes (*Hoxa5/7/9/10, Meis1* and *Pbx3*) highlighted with arrows. Expression in *Mir142*^–/–^GMPs is shown for comparison*.* Columns represent normalized expression values from each sample isolated from individual mice (WT + IDH2^R140Q^ (*n* = *4*), *Mir142*^–/–^ + IDH2^R140Q^ (*n* = *6*), and *Mir142*^–/–^* (n* = *3)*). (**f**) Heatmap of most significantly differentially expressed genes in CD34^+^Mac1^+^ myeloblasts and Mac1^+^(CD34^–^) myeloid cells isolated from *Mir142*^–/–^ mice and *Mir142*^–/–^ + IDH2^R140Q^ recipients (GFP + cells). (**g**) Expression of differentially expressed *Hoxa* cluster genes and *Meis1* in CD34^+^Mac1^+^ myeloblasts and Mac1^+^(CD34^–^) myeloid cells in bone marrow of *Mir142*^*–/–*^ mice (*padj* < 0.05, *n* = 2). (**h**) Expression of differentially expressed *Hoxa* cluster genes and *Meis1* in *Mir142*^–/–^ + IDH2^R140Q^ GMPs, CD34^+^Mac1^+^ myeloblasts and mature Mac1^+^(CD34^–^) cells (*padj* < *0.05, n* = *2*). (**i**) Expression of homeobox genes including *Hoxa* cluster genes (*Hoxa5/7/9/10*), *Meis1* and *Pbx3,* and the HOX regulator *Ash1l* in WT + CTL (*n* = *3*), WT + IDH2^R140Q^ (*n* = *4*), *Mir142*^–/–^ + IDH2^R140Q^ (*n* = *6*) and *Mir142*^–/–^ (n =3) GMPs. (**j**) CFC assay with WT and *Mir142*^–/–^ HSPCs co-transduced with IDH2^R140Q^ and either an shRNA targeting *Ash1l* (shAsh1l) or non-targeting control (shNeg) (*n* = *2*). Statistical significance assessed by unpaired two-tailed *t* test*.*

To investigate the mechanism of cooperation between *Mir142* loss-of-function and IDH2^R140^^Q^, we performed RNA-seq analysis on myeloid progenitors (GMPs) isolated from WT and *Mir142*^−/−^ mice, as well as WT + CTL, WT + IDH2^R140Q^, and *Mir142*^−/−^ + IDH2^R140Q^ GMPs isolated from transplant recipients (Fig. 6a, Supplementary Fig. 2a–c, Supplementary data files 1–3).

Predicted targets of miR-142-3p were highly enriched within the genes upregulated in *Mir142*^−/−^ and double mutant *Mir142*^−/−^ + IDH2^R140^^Q^ cells, accounting for 16% and 17% of upregulated genes respectively (Fig. 6a–c, Supplementary data file 1). miR-142-3p has been reported to produce two isomirs (miR-142-3p.1 and miR-142-3p.2) which differ by one nucleotide at the 5′ end^31,32^. Predicted targets of both isomirs were highly enriched within the upregulated genes in both *Mir142*^−/−^ and *Mir142*^−/−^ + IDH2^R140Q^ cells compared to WT or WT + IDH2^R140Q^ controls respectively, (Fig. 6a–c, Supplementary Fig. 2b,h) indicating that both isomirs are functional in myeloid progenitors.

Gene set enrichment analysis revealed a strong enrichment for a mutant-NPM1 leukemia associated signature within the genes upregulated in the double mutant (*Mir142*^−/−^ + IDH2^R140^^Q^) GMPs, including several homeobox genes and HOX cofactors^33^ (Fig. 6d,e). The *Hoxa* cluster genes—*Hoxa5, Hoxa7, Hoxa9,* and *Hoxa10*—and genes encoding the leukemogenic *Hox* co-factors, *Meis1* and *Pbx3,* were all upregulated in double mutant *Mir142*^−/−^ + IDH2^R140Q^ GMPs compared to their WT + IDH2^R140Q^ counterparts (Fig. 6e, Supplementary data file 2). In addition, *Ash1l* and *Ctnnb1* (β-catenin), which have previously been shown to mediate expansion of myeloid progenitors and promote leukemic transformation in MLL/HOX-driven leukemias^34–36^, were both significantly upregulated in *Mir142*^−/−^ + IDH2^R140Q^ GMPs (Fig. 6c,i, Supplementary Fig. 2d and Supplementary data file 2). *Ash1l, Ctnnb1, Hoxa7,* and *Hoxa9* were also upregulated in the single mutant *Mir-142*^−/−^ GMPs compared to WT, but the key co-factors *Meis1* and *Pbx3* were not, potentially explaining why these mice failed to develop leukemia (Supplementary data file 1).

To understand the origin of the aberrantly expanded CD34^+^ Mac1^+^ myeloblast population we isolated this population, together with the CD34^-^ Mac1^+^ cells from both the leukemic *Mir142*^−/−^ + IDH2^R140^^Q^ recipients and the single mutant *Mir142*^−/−^ mice, and performed RNA-seq (Fig. 6f). This analysis revealed that the *Mir142*^−/−^ CD34^+^ Mac1^+^ myeloblasts expressed very high levels of *Hoxa* cluster genes compared to the more differentiated CD34^-^ Mac1^+^ myeloid cells (Fig. 6g). Furthermore, in leukemic *Mir142*^−/−^ + IDH2^R140Q^ recipients, both the CD34^+^ Mac1^+^ and CD34^-^ Mac1^+^ myeloid cells expressed high levels of *Hoxa* genes, similar to the levels of expression in GMPs (Fig. 6h). The expansion of myeloid populations in *Mir142*^−/−^ animals and in *Mir142*^−/−^ + IDH2^R140Q^ leukemias is therefore likely to be caused by failure to downregulate *Hoxa* cluster genes during myeloid differentiation.

Given our observations, and the oncogenic nature of IDH2 mutations, we initially expected that *Hoxa* genes would be activated by IDH2^R140^^Q^; however, to our surprise, we discovered that *Hoxa5*, *Hoxa7 and Hoxa9* were in fact expressed at a significantly lower level in IDH2^R140Q^ GMPs (Fig. 6i and Supplementary data file 3). This effect was specifically observed in myeloid progenitors and not in KLS HSPCs (Supplementary Fig. 2e). Crucially, this suppression of *Hoxa* cluster expression was alleviated in *Mir142*^−/−^ + IDH2^R140Q^ GMPs (Fig. 6i), and *Hoxa10* was significantly upregulated, revealing that loss of *Mir142* function cooperates with IDH2^R140Q^ by preventing downregulation of these key regulators of leukemic transformation. This led us to speculate that it may be a pre-requisite for mutations that collaborate with IDH2^R140^ mutants to counteract *Hoxa* cluster repression.

### *Hoxa* cluster activation through a *Mir142*-*Ash1l* axis is required for expansion of myeloid progenitors

We then asked how *Mir142* loss-of-function promotes *Hoxa* cluster activation, and whether upregulation of *Hoxa* genes is required for the expansion of myeloid progenitors in the *Mir142*^−/−^ + IDH2^R140^^Q^ cells. We first identified predicted miR-142-3p targets that were differentially expressed both in *Mir142*^−/−^ and in *Mir142*^−/−^ + IDH2^R140Q^ GMPs (Fig. 6c). Of these predicted targets, an obvious candidate was *Ash1l,* which encodes a histone H3K36 methyltransferase belonging to the MLL/Trithorax family, which has important functions in promoting *HOX* gene expression in both normal and leukemic cells^36–39^. Targetscan predicted four miR-142-3p target sites in the *Ash1l* 3′UTR—two of which are highly conserved^40^. *Ash1l* has been previously shown to be a direct target of miR-142-3p, and depletion of *Ashl1* using a gene trap allele prevented upregulation of *Hoxa9* in *Mir142*^−/−^ bone marrow^17,41^.

We therefore investigated whether the knockdown of *Ash1l* could ameliorate the effect of *Mir142* loss-of-function in myeloid progenitors using an shRNA targeting *Ash1l* (Fig. 6j, Supplementary Fig. 2f,g). *Mir142*^−/−^ + IDH2^R140^^Q^ HSPCs produced an increased proportion of myeloid colonies compared to WT + IDH2^R140Q^ HSPCs (Fig. 6j). However, knockdown of *Ash1l* partially suppressed this increase in myeloid colony formation, while having no significant effect on WT + IDH2^R140Q^ cells or *Mir142*^−/−^ + CTL cells (Fig. 6j and Supplementary Fig. 2g). This data is consistent with a compensatory antagonistic model of cooperation between these mutations, wherein *Mir142* loss-of-function counteracts the suppression of *HOX* gene expression by IDH2^R140Q^, through the upregulation of *HOX* regulatory factors such as *Ash1l*, thus unlocking leukemic transformation of IDH2^R140Q^-mutated myeloid progenitors.

## Discussion

AML is a highly intractable cancer, and the prognosis for AML patients has improved little in recent years despite significant increases in our understanding of the genetic and epigenetic complexity of this disease. The key to developing better treatments will be to understand how the different recurrent mutations identified in AML patients collaborate to drive development of overt leukemia. However, this will require careful modelling of each these co-occurring mutations, individually and in combination, to identify the underlying mechanisms of their cooperative leukemogenic effects.

Here, we have modelled the synergy between two disease-relevant, co-occurring mutations in an in vivo setting, and used gene expression analysis of defined progenitor subsets to investigate how they cooperate to drive leukemogenesis. The *MIR142* mutations identified in AML patients, exclusively co-occurred with IDH mutations in the TCGA cohort, and in one patient from the cohort of AML and MDS patients described by Thol et al.^1,8^. Our analysis revealed that, counterintuitive to its oncogenic role, IDH2^R140^^Q^ actually leads to lower expression of the pro-leukemic *Hoxa* cluster genes in GMPs but this is counteracted by the loss of *Mir142*. Recipients of *Mir142*^−/−^ + IDH2^R140Q^ double mutant HSPCs developed a myeloid leukemia characterized by an expansion of immature myeloid cells and a profound inhibition of normal hematopoiesis resulting in peripheral cytopenia, that was not induced by either *Mir142* loss-of-function or IDH2^R140Q^ alone; thus demonstrating that loss of *Mir142* unlocks the leukemogenic potential of IDH2^R140Q^. Knockdown experiments suggested that this effect is likely to be mediated, at least in part, by the upregulation of the MLL/Trithorax-family histone methyltransferase ASH1L, a known regulator of *HOX* gene expression in both normal and leukemic cells.

The mutations affecting *MIR142* in AML were all located in the seed region of miR-142-3p suggesting that they cause loss of targeting of canonical *MIR142* targets and we and others have confirmed this using luciferase reporter assays^8,17^. However, it is possible that the mutated seed can recognise new targets and could contribute to the impact of these mutations. However, since the mutations are located at 5 different positions, each would create a different neo-target making it unlikely that they have convergent effects. It is therefore likely that loss of function is the main cause of the leukemogenic function of these mutants as supported by the finding by us and others that *Mir142* loss synergises with IDH2 mutations to promote leukemogenesis in mice^17^.

Activation of the *HOX* cluster is a key feature of many types of AML, and *HOXA9* is a potent oncogene^42–46^. Nevertheless, *HOXA9* requires the upregulation of the homeodomain-containing co-factors, *MEIS1* or *PBX3*, to promote leukemogenesis^47–50^. While *Hoxa* genes were upregulated in *Mir142*^−/−^ GMPs, *Meis1* and *Pbx3* were not, consistent with the failure of these mice to develop leukemia. However, *Meis1* and *Pbx3* were activated in *Mir142*^−/−^ + IDH2^R140^^Q^ leukemic GMPs, suggesting that synergistic activation of these co-factors may contribute to the leukemogenic effect of our combined mutations in *Mir142* and *IDH2*. Our data, therefore, points to a dual mechanism underpinning the inter-dependence of *Mir142* loss-of-function and IDH mutations in AML: antagonistic regulation of *HOXA* cluster expression and mutual activation of homeobox co-factors.

The antagonistic effect of *Mir142* and IDH on *HOX* gene expression may have wider implications outside of this small subgroup of *Mir142* mutant AML, as it suggests that IDH mutations—which are one of the most common aberrations in AML—may stringently require *HOX* activation for their leukemogenic consequences to be realized. Notably, *IDH* mutations frequently co-occur with NPM1 mutations, which are strongly associated with a *HOX* gene expression signature^33,51^, and *HOX/Meis1* overexpression is required to maintain the leukemic state in NPM1 mutant cells^52^. Therefore mutations in NPM1 and *Mir142* may provide a convergent role in IDH-mutant leukemias, namely to activate *HOX* gene expression.

In summary, our findings provide a new framework for understanding genetic cooperativity in cancer. The prevailing concept is that each and every consequence of a cancer-associated mutation directly contributes to increased risk of tumour formation or, at least, is neutral in its current context. However, multistep tumorigenesis and clonal evolution may be better understood in terms of less coherent outcomes from mutations. We propose that cancer-associated variants can act incoherently, with both positive and negative effects on oncogenesis, resulting in the co-selection of mutations that alleviate their tumour-suppressive properties. It will be interesting to identify further examples of this type of genetic complementation between co-occurring driver mutations. This model provides a compelling rationale for understanding the context-dependency of so-called driver mutations which are often necessary, but not sufficient, to drive oncogenesis.

## Methods

### Animals

Mice were maintained in specific pathogen-free conditions and all experimental protocols were performed in accordance with United Kingdom Home Office regulations. Two mouse models with *Mir142* deletions were used in this study. The B6-*Mir142*^*em2Card*^ strain carries a 26 bp deletion of the miR-142-3p sequence generated by CRISPR-Cas9 editing (provided by Masatake and Kimi Araki, Centre for Animal Resources and Development (CARD), Kumamoto University, Japan). The *Mir142*^*HOJ2*^ line carries a 276 bp deletion of the *Mir142* locus generated through germline Cre-mediated recombination of a floxed *Mir142* allele (provided by Graham Lord, King’s College London, UK)^14^. In the transplantation studies, 8–16 week old, male and female B6.SJL-*Ptprc*^*a*^* Pepc*^*b*^/BoyJ (CD45.1) mice were used as recipients and to provide support/competitor cells to ameliorate effects of myeloablation. Maintenance of the B6-*Mir142*^*em2Card*^ and CD45.1 mice, and all transplantation experiments were performed at University College London (UCL) under UK Home Office Project License PPL:70/8143. The *Mir142*^*HOJ2*^ line was maintained at Kings College London under PPL:70/7869.

### Lentiviral vector production

VSV-G pseudotyped lentiviral vectors were produced by transient transfection of CSI lentiviral plasmids with psPAX2 and pMD2.G packaging plasmids into sub-confluent HEK293T cells using FuGene 6 (E2691, Promega, Madison, WI, USA). Viral supernatants were collected and concentrated by centrifugation at 50,000*g* for 2.5 h at 4 °C before being resuspended in IMDM. Viral titre was estimated by transducing HEK293T cells with serially diluted vector and quantifying the proportion of GFP + cells by flow cytometry.

### Luciferase reporter assay for miR-142-3p activity

HEK293T cells were transduced at matched MOI with lentiviral vectors expressing either WT or mutant *MIR142* with a GFP reporter, or empty vector control. GFP + cells were sorted after 3 days and transfected with the miR-142-3p luciferase reporter. Luciferase assays were then performed following the manufacturer’s protocol using Dual-Glo Luciferase Assay system (Promega, E2920) and luminescence measured on the Varioskan LUX (N16045, Thermo Fisher Scientific, Waltham, MA USA).

### Transplantation assays

Whole bone marrow (5 × 10^5^ cells) or c-Kit^+^ Lineage^−^ Sca1^+^ (KLS) HSPCs (1 × 10^4^ cells) from CD45.2 + donors were mixed with 1 × 10^5^ CD45.1 + whole bone marrow support/competitor cells and injected into the tail vein of irradiated CD45.1 recipient mice. Irradiation was performed using an X-ray irradiator to deliver 800 cGy as a split dose.

For leukemia modelling, 1 × 10^4^ KLS cells per recipient were FACS-purified from CD45.2 + wild type or *Mir142*^−/−^ donors, and resuspended in StemSpan serum-free expansion medium (Stem Cell Technologies, Vancouver, BC, Canada), supplemented with 100 units/mL penicillin, 100 ng/ml streptomycin, 200 ng/mL SCF, 20 ng/mL Flt3-Ligand, and 20 ng/mL TPO. Cells were pre-incubated for 1–2 h at 37 °C in the above cytokines and transduced with lentiviral vector (either SFFV-IDH2^R140^^Q^-IRES-GFP or empty SFFV-IRES-GFP control vector) overnight at 37 °C, 5% CO_2_. The cells were then washed and co-injected with 2 × 10^5^ CD45.1 whole bone marrow support/competitor cells into irradiated (800 cGy) CD45.1 recipient mice. Animals that showed no engraftment of donor cells were excluded from further analysis. Mice exhibiting declining health status were sacrificed and tissues taken for analysis.

### Colony forming cell (CFC) assays

KLS cells were FACS-purified from bone marrow samples, seeded into 1.5 mL of Methocult M3434 (Stem Cell Technologies) and plated into 35 mm non-coated plates (430,588, Corning Incorporated, Corning, NY, USA). Plates were incubated for 10–14 days at 37 °C, 5% CO_2_. Colonies produced were counted and classified.

### RNA-seq library preparation

RNA was extracted from KLS, GMP, CD34^+^Mac1^+^ and CD34^-^Mac1^+^ cells, and RNA-seq libraries were prepared using SMART-Seq v4 Ultra Low Input RNA Kit for Sequencing (634,891, Takara Bio, Kusatsu, Japan), and the Nextera XT library preparation kit (FC-131-1096, Illumina, San Diego, CA, USA). Libraries were then sequenced on an Illumina NextSeq 500**.**

### Statistical analysis

Statistical analyses including assessments of significance, variance and normality were performed using Prism 8 software (GraphPad, San Diego, CA, USA). Results are expressed as the mean ± standard error of the mean (SEM) unless otherwise indicated. Significant differences between experimental groups were determined by a two-tailed Mann–Whitney test unless otherwise indicated. Survival data was analyzed using the Mantel-Cox log-rank test. *P* values shown as follows: *P < 0.05, **P < 0.005, ***P < 0.0005, ****P < 0.0001.

## Supplementary information

Supplementary Information 1.

Supplementary Information 2.

Supplementary Information 3.

Supplementary Information 4.

## Data Availability

RNA-seq data generated during the current study was deposited in Array Express (https://www.ebi.ac.uk/arrayexpress/) with accession number: E-MTAB-8042.
