# Author Correction: *Mir142* loss unlocks IDH2^R140^-dependent leukemogenesis through antagonistic regulation of *HOX* genes

**DOI:** 10.1038/s41598-021-84578-y

**Published:** 2021-03-23

**Authors:** A. Marshall, J. Kasturiarachchi, P. Datta, Y. Guo, E. Deltcheva, C. James, J. Brown, G. May, N. Anandagoda, I. Jackson, J. K. Howard, E. Ghazaly, S. Brooks, A. Khwaja, M. Araki, K. Araki, D. Linch, G. M. Lord, T. Enver, R. Nimmo

**Affiliations:** 1 UCL Cancer Institute, University College London, London, UK; 2 School of Immunology and Microbial Sciences, King’s College London, London, UK; 3 School of Life Course Sciences, King’s College London, London, UK; 4 Centre for Haemato‑Oncology, Barts Cancer Institute, Queen Mary University of London, London, Queen UK; 5 Institute of Resource Development and Analysis, Kumamoto University, Kumamoto, Japan; 6 Faculty of Biology, Medicine and Health, University of Manchester, Manchester, UK; 7 Present Address: Medicines and Healthcare Products Regulatory Agency (MHRA), London, UK; 8 Present Address: Oxford Biomedica (UK) Ltd, Windrush Court, Transport Way, Oxford, OX4 6LT UK; 9 Present Address: The Institute of Cancer Research, London, UK

Correction to: *Scientific Reports*, 10.1038/s41598-020-76218-8, published online 10 November 2020

In the original version of this Article, R. Nimmo was incorrectly affiliated with ‘School of Immunology and Microbial Sciences, King’s College London, London, UK.’ The correct affiliations are listed below.

UCL Cancer Institute, University College London, London, UK.

Present address: Oxford Biomedica (UK) Ltd, Windrush Court, Transport Way, Oxford OX4 6LT, UK.

This error has now been corrected in the PDF and HTML versions of the Article.

