# Supplementary material for: Mir142 loss unlocks IDH2R140-dependent leukemogenesis through antagonistic regulation of HOX genes
Source: Sci Rep. 2020 Nov 10;10:19390. doi: 10.1038/s41598-020-76218-8 (PMC7656267; doi:10.1038/s41598-020-76218-8)
Supplement: Supplementary file 4 — Supplementary Information 4. [file 41598_2020_76218_MOESM4_ESM.pdf]

Supplementary Information

Mir142 loss unlocks IDH2^{R140}-dependent leukemogenesis through antagonistic regulation of *HOX* genes

Authors

Marshall A[1], Kasturiarachchi J[1], Datta P, Guo Y, Deltcheva E, James C, Brown J, May G, Anandagoda N, Jackson I, Howard J.K, Ghazaly E, Brooks S, Khwaja A, Araki M, Araki K, Linch D, Lord G.M, Enver T, Nimmo R*.

[1]These authors contributed equally to this work.

*Corresponding author.

Supplementary Materials and Methods

Plasmid generation

To generate the lentiviral SFFV-IDH2^{R140Q}-IRES-GFP vector, cDNA encoding mutant IDH2^{R140Q} was inserted into a unique BamHI site in the multiple cloning site of the CSI (SFFV-IRES-GFP) lentiviral plasmid. Lentiviral vectors expressing wildtype or variant forms of *MIR142*, were generated by cloning the miRNA into the CSI plasmid downstream of the GFP. gBlock oligonucleotides encoding wildtype or mutant *MIR142* pre-miR sequences (mut55 A>G, mut57 U>C and mut58 G>C) with additional flanking sequences, were synthesized by Integrated DNA Technologies (Leuven, Belgium) and cloned into NotI and SbfI sites in the CSI plasmid. To generate the miR-142-3p luciferase reporter, an oligonucleotide containing three copies of the canonical bulged miR-142-3p target sequence was cloned into the pmirGLO Dual-Luciferase miRNA Target Expression Vector (Promega, E1330).

Relative quantification of expression by qRT-PCR

RNA was extracted from cells using TRI reagent (Sigma Aldrich, 93289) according to the manufacturer's protocol and DNase treated. For miRNA quantification, reverse transcription was performed using the TaqMan MicroRNA Reverse transcription kit (Thermo Fisher Scientific, 4366596) followed by qPCR using TaqMan MicroRNA Assays (Thermo Fisher Scientific, 4427975) according to the manufacturer's protocol. Assays used for analysis were miR-142-3p (Assay ID: 000464) and U6 endogenous control (Assay ID: 001973). For quantification of *Ash1l* expression, reverse transcription was performed using the SuperScript III First-Strand Synthesis System (Thermo Fisher Scientific, 18080051) and TaqMan Gene Expression Assays (Thermo Fisher Scientific, 4331182). Assays used for analysis were *Ash1l* (Assay ID:

Mm00467322_m1) and *Actb* endogenous control (Mm02619580_g1). For quantification of $IDH2^{R140Q}$ expression, the SuperScript III First-Strand Synthesis System (Thermo Fisher Scientific, 18080051) and SYBR Green PCR mastermix (Thermo Fisher Scientific, 4309155) were used, with primers to mouse *Gapdh* and the following transgene-specific primers:

IDH2CSI_F2 ctggatgggaaccaagacct

IDH2CSI_R2 gggatccacgcgtctactg

Quantification of 2-hydroxyglutarate by mass spectrometry

Washed cell pellets were extracted by adding 300µl of 80% methanol and placed on ice for 30 minutes. After centrifugation for 5 minutes (10,000g, 4°C), supernatant was transferred and dried under Savant speed vac (Thermo Fisher Scientific). The dried extracts were reconstituted in 100 µl of 10% acetonitrile (+0.1% formic acid) and 10 µl were injected directly into the LC-MS/MS system. Separation was achieved on an UPLC system (Accela system, Thermo Scientific) equipped with an ACE Ultracore 2.5 µm, Super C18, 100 × 2.1 mm column (Hichrom, UK). Isocratic elution was employed using a mobile phase of 0.1% formic acid in water (70%) and 0.1% formic acid in acetonitrile (30%) for 6 minutes, all at a flow rate of 250 µl/min. Triple-stage-quadrupole mass spectrometry (TSQ Vantage, Thermo Fisher Scientific) equipped with a heated electrospray ion source was used for mass detection. Samples were analyzed in the Multiple Reaction Monitoring (MRM) negative ion ionization mode was employed at a spray voltage of 3000V. Nitrogen was used as sheath and auxiliary gas at a flow rate of 50 and 20 arbitrary units, respectively. Argon was used as collision gas with pressure of 1.5 mTorr. The optimum transitional daughter ions mass and collision energy for 2-HG was: -ve m/z 147.0 $\rightarrow$ 129.1 (collision energy 12

V). Data acquisition and chromatography analysis was carried out using Xcalibur chromatography software version 2.2 from Thermo Fisher Scientific. The method was found to be linear between 0.1 and 10µg/ml (R^2 for weighted $1/x^2$ linear regression > 0.97) and recovery was 83.4 ± 8.3%. Intra-day and Inter-day imprecision were < 2.9% and 10.2%, respectively and inaccuracy was < 9.0% for all quality control levels.

Processing of mouse tissues

For bone marrow extraction, leg and hip bones were crushed in a pestle and mortar and cell suspensions filtered using 40 µm filters. Spleen and liver were diced and passed through a 70 µm filter. Cell suspensions were then centrifuged and resuspended in 1x RBC lysis buffer (BioLegend, San Diego, CA, USA, 420301) for 10-15 minutes on ice. Cells were centrifuged, resuspended in PBS containing 2% heat-inactivated FBS and 2mM EDTA (FACS buffer), filtered, and stained with the appropriate antibody panels. All centrifugation steps were carried out at 400g, 4°C for 5 minutes.

Peripheral blood analysis

Peripheral blood was collected in EDTA-coated tubes and blood counts were obtained using a Sysmex XP-300™ Automated Hematology Analyzer. Red blood cells were lysed using 1xRBC lysis buffer (BioLegend, 420301) for 10-15 minutes at room temperature. Cells were centrifuged, resuspended in FACS buffer, filtered and stained with appropriate antibody panels. All centrifugation steps were carried out at 400g, 4°C for 5 minutes.

Flow cytometry analysis and cell sorting

Flow cytometry analysis was performed on a Gallios flow cytometer (Beckman Coulter) using Kaluza acquisition and analysis software. FACS sorting was performed on a BD FACSAria III. For sorting KLS and GMP populations, c-Kit$^+$ cells were first enriched using CD117 MACS beads (Miltenyi Biotec, Bergisch Gladbach, Germany, 130-091-224). Progenitor populations were defined by surface markers: KLS (Kit$^+$ Lin$^-$ Sca1$^+$), CMP (Kit$^+$ Lin$^-$ Sca1$^-$ CD34$^+$ FcYR$^{lo/-}$), GMP (Kit$^+$ Lin$^-$ Sca1$^-$ CD34$^+$ FcYR$^+$), MEP (Kit$^+$ Lin$^-$ Sca1$^-$ CD34$^-$ FcYR$^-$), CLP (Lin$^-$ IL7R$^+$ Sca1^{lo} Kit^{lo}). HSC/MPP subsets were defined by either CD34/Flt3 [1] or SLAM markers (CD150/CD48) [2]: LT-HSC (CD34$^–$Flt3$^–$ KLS), ST-HSC (CD34$^+$ Flt3$^–$ KLS), LMPP (CD34$^+$ Flt3$^+$ KLS), SLAM HSC (CD150$^+$ CD48$^–$ KLS), SLAM MPP (CD150$^–$ CD48$^–$ KLS) and SLAM HPC1 (CD150$^–$CD48$^+$ KLS). The following antibodies were used for flow cytometry: Sca1 (D7), c-Kit/CD117 (2B8), FcGR(CD16/32) (93), CD34 (RAM34), CD45.2 (104), CD45.1 (A20), CD150 (TC15-12F12.2),CD48 (HM48-1), Flt3 (A2F10), IL7R (A7R34), CD71 (RI7217), CD41 (MWReg30), CD3 (145-2C11), CD8 (53-6.7), CD4 (GK1.5), B220 (RA3.6B2), Ter119 (TER119), Gr1 (RB6-8C5), CD11b/Mac1 (M1/70). Antibodies were obtained from either BioLegend or eBioscience/Thermo Fisher Scientific. Hoechst 33258 (0.5 μg/ml) or Propidium Iodide (0.2 μg/ml) were used to asses cell viability. Cytospins were performed on sorted CD34$^+$ Mac1$^+$ cells by spinning at 500rpm for 5 minutes in a Shandon CytoSpin and stained with May-Grünwald-Giemsa (Sigma Aldrich, St. Louis, MO, USA).

RNA-seq library preparation

KLS, GMP, $CD34^+Mac1^+$ and $CD34^-Mac1^+$ cells were FACS-purified and RNA extracted using TRI Reagent (Sigma Aldrich, 93289). RNA quality was confirmed on an Agilent Bioanalyzer using the RNA 6000 Pico Kit (Agilent, Santa Clara, CA, USA, 5067-1513) and quantified using either the Qubit RNA HS Assay Kit (Thermo Fisher Scientific, Q32855) or the Bioanalyzer for samples with low concentration. RNA was then reverse transcribed and amplified using SMART-Seq v4 Ultra Low Input RNA Kit for Sequencing (Takara Bio, Kusatsu, Japan, 634891), and libraries generated using Nextera XT library preparation kit (Illumina, FC-131-1096). Libraries were checked using Bioanalyzer High Sensitivity DNA Kit (Agilent, 5067-4626), quantified using Qubit dsDNA HS Assay kit (Thermo Fisher Scientific, Q33231) and sequenced on an Illumina NextSeq 550 using the 150 cycle High Output v2 kit (FC-404-2002) with 2x75bp read length.

Bioinformatic analysis of RNA-seq

Fastq files from paired RNA sequencing reads were run through a quality control and adaptor trimming protocol using Trim Galore, a wrapper script running FastQC [3] and cutadapt [4]. The trimmed reads where then aligned to reference indices generated from the Genome Reference Consortium Mouse Build 38 (https://www.ncbi.nlm.nih.gov/grc) using HISAT2 [5]. Aligned files were run through quality control and counts for reads aligning to known genes were determined using QoRTs [6]. Fragments per kilobase of exon per million reads mapped (FPKMs) where calculated using Cuffllinks [7,8]. The R package DESeq2 [9] was used to analyze differential gene expression and modified log fold changes were used to create ranked lists for gene set enrichment analysis [10,11]. Gene set enrichment analysis was performed against lists in the Molecular Signatures Database (MsigDB) 3.0 [12]. In

addition, a custom gene set containing targets of both miR-142-3p.1 and miR-142-3p.2 isomirs (as predicted by Targetscan), was generated to determine enrichment of miR-142-3p targets within the differentially expressed genes.

Lentiviral shRNA for *Ash1l* knockdown

Lentiviral constructs expressing shRNAs targeting *Ash1l* (ULTRA-3233179) or a non-targeting control (ULTRA-NT#4), were obtained from TransOMIC technologies. In the pZip-SFFV-turboRFP-Puro vector, the shRNA is expressed from an optimised UltramiR backbone and co-expressed with RFP. *Ash1l* knockdown was validated in the HPC5 murine hematopoietic progenitor cell line. FACS-purified KLS cells were then co-transduced with the shRNA pZip vector and the SFFV-IDH2^{R140Q}-IRES-GFP vector. Double transduced GFP+RFP+ cells were sorted by FACS, and CFC assays performed as described above.

Supplementary data files

Supplementary data files are also provided, containing DEGs (*padj<0.05*) for the following sample comparisons: *Mir142*$^{-/-}$ vs. WT GMPs (Supplemental data file 1), *Mir142*$^{-/-}$ + IDH2^{R140Q} vs. WT + IDH2^{R140Q} GMPs (Supplemental data file 2) and WT + IDH2^{R140Q} vs. WT + CTL GMPs (Supplemental data file 3).

Supplementary References

1. Adolfsson, J. *et al.* Identification of Flt3+ lympho-myeloid stem cells lacking erythro-megakaryocytic potential a revised road map for adult blood lineage commitment. *Cell* **121**, 295–306 (2005).
2. Kiel, M. J. *et al.* SLAM family receptors distinguish hematopoietic stem and

progenitor cells and reveal endothelial niches for stem cells. *Cell* **121**, 1109–21 (2005).

3. Andrews, S. FastQC A Quality Control tool for High Throughput Sequence Data. *http://www.bioinformatics.babraham.ac.uk/projects/fastqc/* (2010).
4. Martin, M. Cutadapt removes adapter sequences from high-throughput sequencing reads. *EMBnet.journal* **17**, 10 (2011).
5. Kim, D., Langmead, B. & Salzberg, S. L. HISAT: a fast spliced aligner with low memory requirements. *Nat. Methods* **12**, 357–360 (2015).
6. Hartley, S. W. & Mullikin, J. C. QoRTs: a comprehensive toolset for quality control and data processing of RNA-Seq experiments. *BMC Bioinformatics* **16**, 224 (2015).
7. Trapnell, C. *et al.* Transcript assembly and quantification by RNA-Seq reveals unannotated transcripts and isoform switching during cell differentiation. *Nat. Biotechnol.* **28**, 511–5 (2010).
8. Roberts, A., Trapnell, C., Donaghey, J., Rinn, J. L. & Pachter, L. Improving RNA-Seq expression estimates by correcting for fragment bias. *Genome Biol.* **12**, R22 (2011).
9. Love, M. I., Huber, W. & Anders, S. Moderated estimation of fold change and dispersion for RNA-seq data with DESeq2. *Genome Biol.* **15**, 550 (2014).
10. Subramanian, A. *et al.* Gene set enrichment analysis: a knowledge-based approach for interpreting genome-wide expression profiles. *Proc. Natl. Acad. Sci. U. S. A.* **102**, 15545–50 (2005).
11. Zhu, A., Ibrahim, J. G. & Love, M. I. Heavy-tailed prior distributions for sequence count data: removing the noise and preserving large differences. *Bioinformatics* (2018). doi:10.1093/bioinformatics/bty895

12. Liberzon, A. *et al.* Molecular signatures database (MSigDB) 3.0. *Bioinformatics* **27**, 1739–1740 (2011).

Supplementary Figures

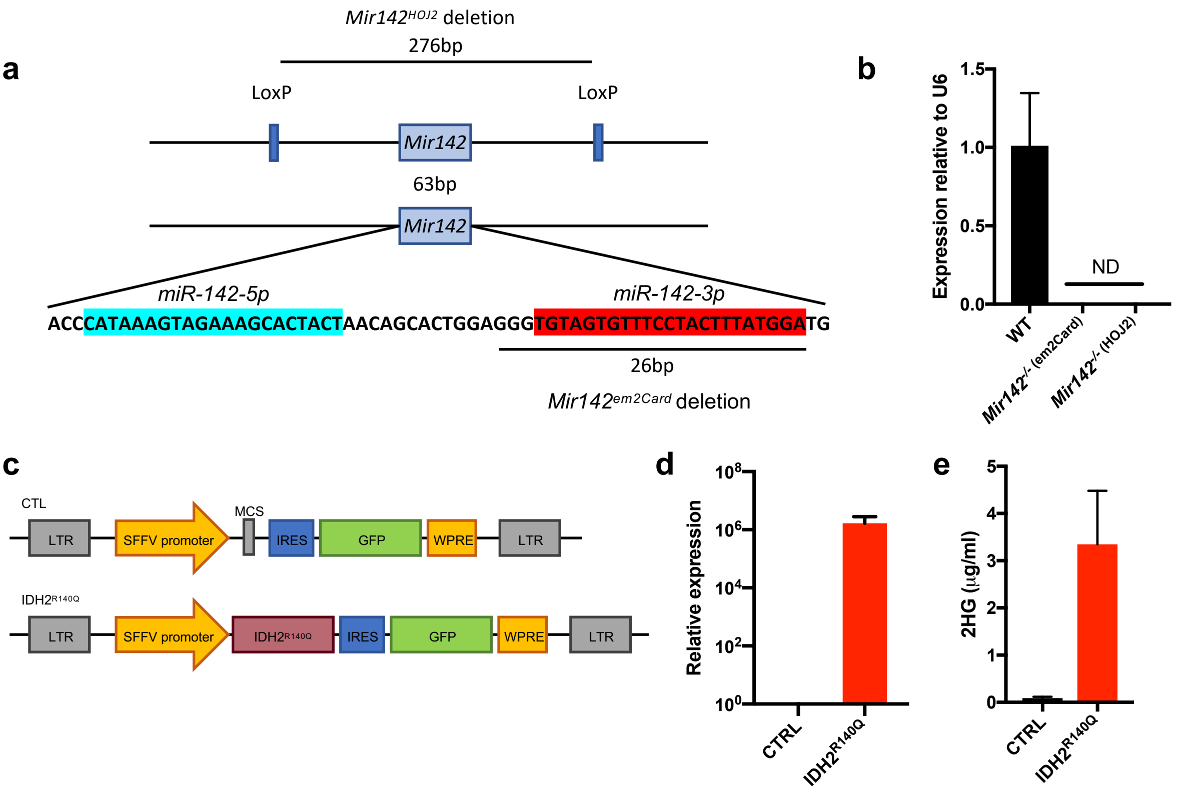

Supplementary Figure 1 (supporting main Figure 2). *Mir142* loss-of-function synergizes with IDH2^{R140Q} to promote myeloid leukemogenesis in mice.

a) Schematic diagram showing the location of the deletions in the two *Mir142* knockout alleles analyzed. Top: *Mir142*^{HOJ2} was generated by Cre-mediated recombination between *LoxP* sites flanking the *Mir142* pre-miR sequence (276bp deletion). Bottom: *Mir142*^{em2Card} was generated using CRISPR-Cas9 to delete only the miR-142-3p sequence (26bp deletion). b) qRT-PCR analysis of miR-142-3p expression in bone marrow from *Mir142*$^{–/–}$ mice. Shown relative to U6 snRNA expression and normalized to WT controls. ND: not detected. c) Schematic diagram showing the lentiviral vector used for expression of IDH2^{R140Q} mutant in murine hematopoietic cells. Top: Empty SFFV-IRES-GFP vector used as control (CTL). Bottom: SFFV-IDH2^{R140Q}-IRES-GFP vector. d) qRT-PCR analysis showing relative expression of IDH2R^{140Q} in bone marrow cells transduced with SFFV-IDH2^{R140Q}-IRES-GFP compared to empty vector control. e) Mass spectrometry analysis of 2-HG levels in cell extracts from IDH2R^{140Q}-expressing bone marrow compared to controls.

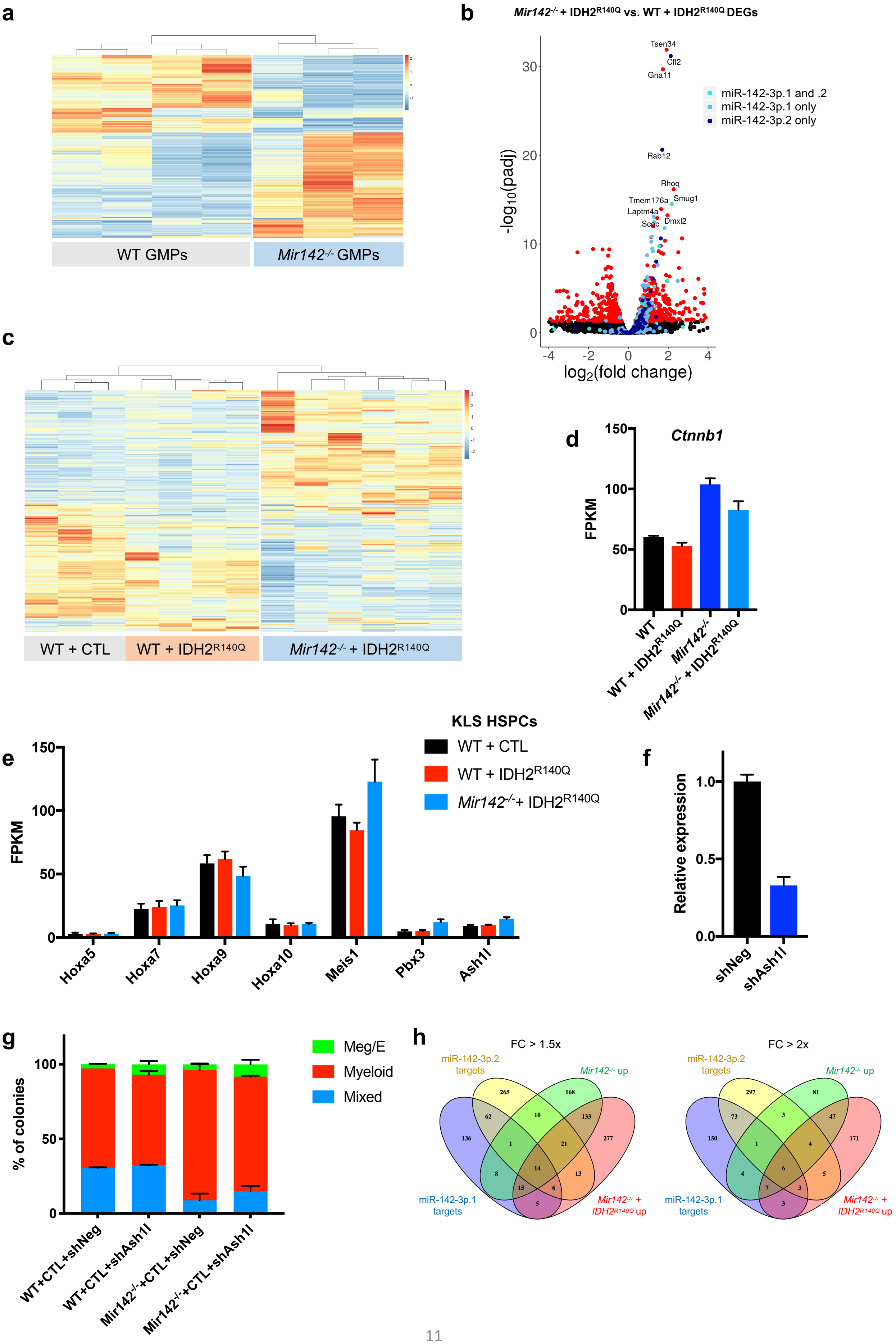
a
WT GMPs
Mir142-/- GMPs
b
Mir142-/- + IDH2R140Q vs. WT + IDH2R140Q DEGs
miR-142-3p.1 and .2
miR-142-3p.1 only
miR-142-3p.2 only
Tsen34
Cfl2
Gna11
Rab12
Rhoq
Tmem176a
Smug1
Laptm4a
Scoc
Dmxl2
-log10(padj)
log2(fold change)
c
WT + CTL
WT + IDH2R140Q
Mir142-/- + IDH2R140Q
d
Ctnnb1
FPKM
WT
WT + IDH2R140Q
Mir142-/-
Mir142-/- + IDH2R140Q
e
KLS HSPCs
WT + CTL
WT + IDH2R140Q
Mir142-/-+ IDH2R140Q
FPKM
Hoxa5
Hoxa7
Hoxa9
Hoxa10
Meis1
Pbx3
Ash1l
f
Relative expression
shNeg
shAsh1l
g
% of colonies
Meg/E
Myeloid
Mixed
WT+CTL+shNeg
WT+CTL+shAsh1l
Mir142-/-+CTL+shNeg
Mir142-/-+CTL+shAsh1l
h
FC > 1.5x
FC > 2x
miR-142-3p.2 targets
Mir142-/- up
miR-142-3p.1 targets
Mir142-/- + IDH2R140Q up

Supplementary Figure 2 (supporting main Figure 6). *Mir142* loss of function upregulates a leukemic *HOX/Meis1/Pbx3* signature and antagonizes IDH2^{R140Q}-dependent silencing of *Hoxa* cluster genes.

a) Heatmap showing most significantly differential genes in GMPs from WT and *Mir142*$^{-/-}$ mice. b) Volcano plot showing differentially expressed genes (DEGs) in *Mir142*$^{-/-}$ + IDH2^{R140Q} vs. WT + IDH2^{R140Q} GMPs (*P<0.05* colored dots). miR-142-3p targets predicted by Targetscan are highlighted in blue. Light blue: miR–142–3p.1 specific targets. Dark blue: miR-142-3p.2 specific targets. Turquoise: targets of both miR-142-3p.1 and miR-142-3p.2 isomirs. c) Heatmap of gene expression in GFP+GMPs isolated from WT + CTL, WT + IDH2^{R140Q} and *Mir142*$^{-/-}$ + IDH2^{R140Q} recipients. Most significantly differential genes from *Mir142*$^{-/-}$ + IDH2^{R140Q} vs. WT + CTL are shown. d) *Ctnnb1* expression in WT, WT + IDH2^{R140Q}, *Mir142*$^{–/–}$ and *Mir142*$^{-/-}$ + IDH2^{R140Q} GMPs (shown as FPKM). e) Expression of homeobox genes including *Hoxa* cluster genes (*Hoxa5/7/9/10*), *Meis1* and *Pbx3,* and the HOX regulator *Ash1l* in WT + CTL, WT + IDH2^{R140Q}, and *Mir142*$^{–/–}$ + IDH2^{R140Q} HSPCs (GFP^{+} KLS population) (shown as FPKM). f) qRT-PCR analysis of *Ash1l* expression in cells transduced with non-targeting control (shNeg) or *Ash1l* (shAsh1l) shRNA constructs. Expression shown relative to *Actb* and normalized to shNeg. g) CFC assay with WT and *Mir142*$^{–/–}$ HSPCs co–transduced with GFP vector and either an shRNA targeting Ash1l (shAsh1l) or non–targeting control (shNeg) *(n=2),* data from the same experiments shown in Figure 6J. h) Venn diagrams showing overlap of miR-142-3p.1 and miR-142-3p.2 targets with DEGs (padj<0.05) from *Mir142*$^{–/–}$ vs. WT GMPs and *Mir142*$^{–/–}$ + IDH2^{R140Q} vs. WT + IDH2^{R140Q} GMPs. All significant DEGs are shown in Fig. 6c, while analysis shown here uses a fold change (FC) cutoff: FC > 1.5 (left) and FC > 2 (right).
